# Huang-Lian Jie-Du decoction: a review on phytochemical, pharmacological and pharmacokinetic investigations

**DOI:** 10.1186/s13020-019-0277-2

**Published:** 2019-12-18

**Authors:** Yiyu Qi, Qichun Zhang, Huaxu Zhu

**Affiliations:** 1 Jiangsu Collaborative Innovation Center of Chinese Medicinal Resources Industrialization, Nanjing University of Chinese Medicine, Nanjing, China; 2 Jiangsu Key Laboratory for High Technology Research of TCM Formulae, Nanjing University of Chinese Medicine, Nanjing, China; 3 Jiangsu Research Center of Botanical Medicine Refinement Engineering, Nanjing University of Chinese Medicine, Nanjing, China; 4 Department of Pharmacology, Pharmacy College, Nanjing University of Chinese Medicine, Nanjing, China

**Keywords:** Huang-Lian Jie-Du decoction, Traditional Chinese medicine, Phytochemical, Parmacological, Pharmacokinetic

## Abstract

Huang-Lian Jie-Du decoction (HLJDD), a famous traditional Chinese prescription constituted by *Rhizoma Coptidis*, *Radix Scutellariae*, *Cortex Phellodendri* and *Fructus Gradeniae*, has notable characteristics of dissipating heat and detoxification, interfering with tumors, hepatic diseases, metabolic disorders, inflammatory or allergic processes, cerebral diseases and microbial infections. Based on the wide clinical applications, accumulating investigations about HLJDD focused on several aspects: (1) chemical analysis to explore the underlying substrates responsible for the therapeutic effects; (2) further determination of pharmacological actions and the possible mechanisms of the whole prescription and of those representative ingredients to provide scientific evidence for traditional clinical applications and to demonstrate the intriguing molecular targets for specific pathological processes; (3) pharmacokinetic feature studies of single or all components of HLJDD to reveal the chemical basis and synergistic actions contributing to the pharmacological and clinically therapeutic effects. In this review, we summarized the main achievements of phytochemical, pharmacological and pharmacokinetic profiles of HLJDD and its herbal or pharmacologically active chemicals, as well as our understanding which further reveals the significance of HLJDD clinically.

## Background

Herbal formula, the most popular therapeutic approach of traditional Chinese medicine (TCM), was recorded in ancient medical literature with fixed herbal components, definite curative effects, and acceptable adverse effects [1].

Huang-Lian Jie-Du decoction (HLJDD) (Oren-gedoku-to in Japanese and Hwangryun-Hae-Dok-Tang in Korean), a well-known classic TCM formula, was first described in Wang Tao’s treatise “Wai Tai Mi Yao” in the Tang dynasty (752 A.D.). It has been a representative prescription for heat-clearing and detoxicating. Heat-clearing is to ameliorate the interior pattern or syndromes of exuberant heat, which is transformed from the process of external pathogens entering the internal organs. The heat is in the form of an elevation in the body temperature above normal or a subjective feeling of feverishness. Detoxicating indicates the measure to reduce the virulence and neutralize the toxicity of pathogens. Here, heat and poison are the forms of pathogens in Chinese medicines. HLJDD shows the ability to dispel the heat and poison and relieve the associated syndromes. This ability is achieved by four common crude herbs, *Rhizoma Coptidis* (RC) (*Coptis chinensis* Franch, Huang Lian), *Radix Scutellariae* (RS) (*Scutellaria baicalensis* Georgi, Huang Qin), *Cortex Phellodendri* (CP) (*Phellodendron amurense* Rupr., Huang Bo), and *Fructus Gradeniae* (FG) (*Gardenia jasminoides* Ellis, Zhi Zi) in a ratio of 3:2:2:3. According to the strict principle of “sovereign, minister, assistant and courier” [2], which was developed from “Huangdi’s Internal Classic” to enhance the effectiveness of Chinese medicinal herbs and to reduce toxics or side effects by combining various kinds of herbs, RC is the sovereign medicine with the action of purging the fire from the heart and middle energizer. RS acts as the ministerial medicine, removing the heat from the lungs and eliminating the fire from the upper energizer. CP purges the fire from lower energizer as the assistant medicine. FG purges the triple energizers and delivers the heat back to its origin as the courier medicine [3]. The whole formula is carefully designed and precise in formation. Xu et al. manufactured four HLJDD variants by leaving one herb out each time and found that the integral formula exhibited the strongest therapeutic effects in the cecal ligation and puncture rats among the four variants [4]. The precise and rigorous herbal combination is believed to be advantageous over single reagent since that various components can hit multiple targets simultaneously and perform synergistic therapeutic actions [5]. Moreover, due to the lack of TCM theories such as the theoretical mechanisms of diseases, researches on decomposed recipes of Chinese herbal compounds find it difficult to reveal the complex interactions between couplet medicines.

**Fig. 1:**
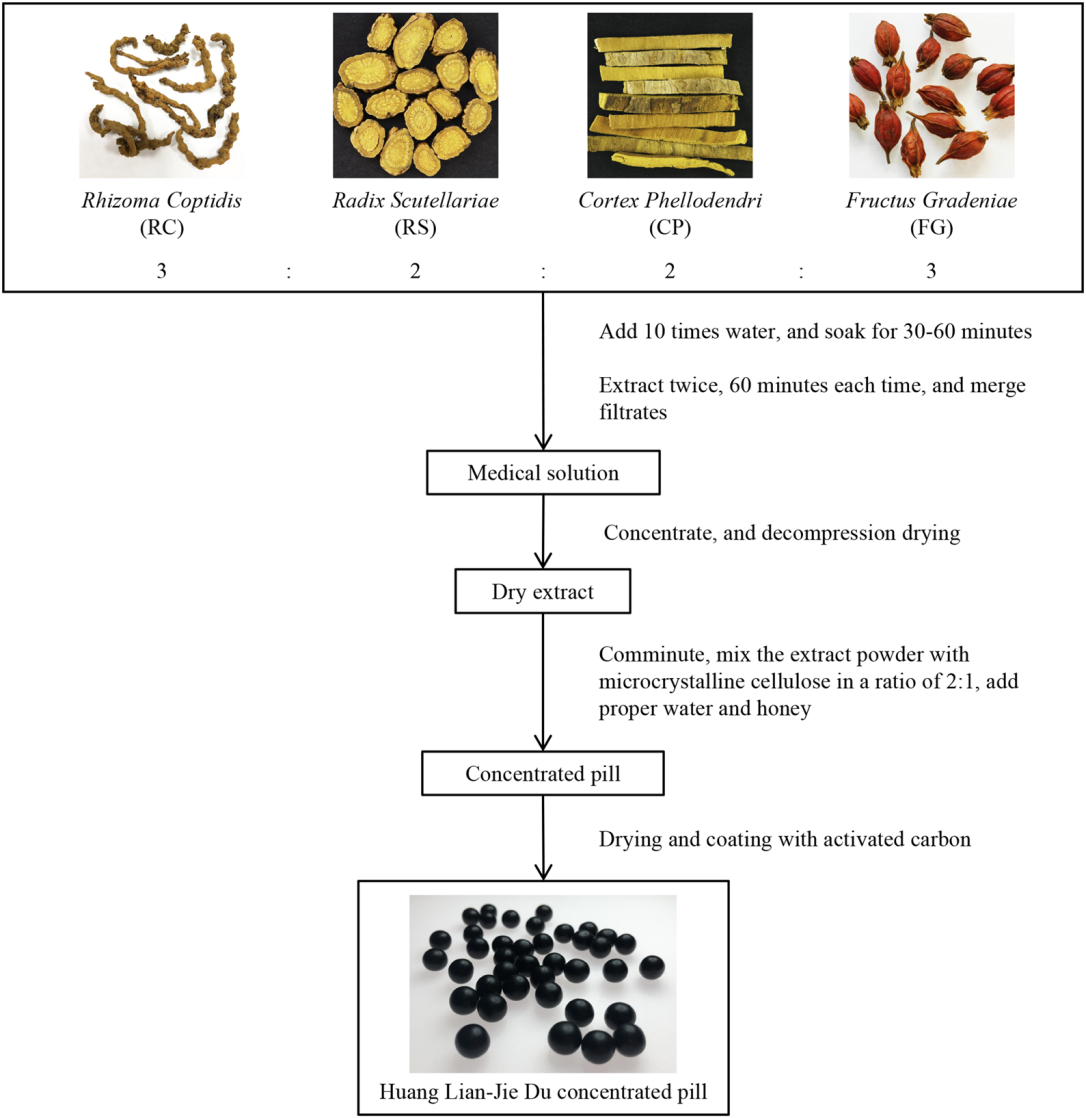
Appearance and processing technology of Huang-Lian Jie-Du concentrated pill

Based on the clinical practice and inheritance of nearly a 1000 years as well as the integration of Chinese and Western medicine, the clinical application of HLJDD has gradually expanded from the diseases and symptoms of TCM to the diseases of Western medicine, and its use has also expanded to other countries besides China. With the remarkable therapeutic effects on removing excess heat and fire toxins, HLJDD plays an important role in the resolution of delirium, internal heat-related mania, insomnia, irritability, dry mouth and throat, heat-induced blood omitting, skin spots, and sore furuncle, according to Medical Secretes of an Official. This formula is also used to treat heat-pathogen-induced pyrostagnant rhinorrhagia, carbuncle, and jaundice as summarized by Prescriptions for Emerent Ref. [6]. At present, HLJDD has been widely used in the clinical practices to treat inflammation, hypertension, gastrointestinal disorders, liver and cerebrovascular diseases [7]. In a clinical study, the addition of HLJDD to yokukan-san (Japanese traditional herbal prescription) exhibited the same efficacy as aripiprazole (antipsychotics) in controlling aggressiveness of an Alzheimer’s type dementia without any significant adverse reaction [8]. Another clinical study indicated that HLJDD was a possible treatment for fever of unknown origin [9]. In China, thin-layer chromatography and microscopy have been employed to establish the quality standard of Huang-Lian Jie-Du pills for decades. The contents of berberine hydrochloride and baicalin have been determined [10]. Additionally, an improved formula of HLJDD in the pill form has acquired the permission of Chinese State Food and Drug Administration to market (drug approval number Z20025356) [11]. The appearance and processing technology of Huang-Lian Jie-Du concentrated pill are shown in Fig. 1. In other Asian countries, HLJDD was approved for palliative cares and atopic dermatitis treatment by Ministry of Health, Labour and Welfare of Japan and Korean Food and Drug Administration [12, 13]. Furthermore, HLJDD has been manufactured as a powdered, freeze-dried water extract by Tsumura Co, Ltd in Japan [9].

More and more clinical application cases have prompted people to explore the potential pharmacological effects and possible molecular mechanisms of HLJDD by modern pharmacology and molecular biotechnology. Modern pharmacological studies indicate that HLJDD exhibits therapeutic actions in various pathological aspects, such as hyperlipidemia [14], tumor [6, 15, 16], arthritis [17–19], sepsis [20–22], cardiac damage [23], liver injury [24, 25], kidney disease [26], cerebral ischemia [27–29], type 2 diabetes mellitus (T2DM) [30, 31], Alzheimer’s disease (AD) [32–34], fungal infection [35] and inflammation [36]. In the meantime, with the deepening of researches and the continuous development of technology, more and more chemical compositions of HLJDD have been discovered. The effects of drugs are based on their chemical composition. This mainstream view holds that the different pharmacological effects and clinical applications of drugs depend on the tissue distribution and concentration of their active ingredients. Therefore, pharmacokinetics (PK) should be adopted to interpret the active substance basis of HLJDD. PK has the characteristics of holistic, comprehensive and dynamic, which is similar to the holistic concept and dialectical treatment of TCM. Although there are numerous researches with positive results on HLJDD, most of them were only performed with a fraction of the total compounds. Hence, it is necessary for us to sum up these past researches which are significant in guilding further researches of HLJDD. In this review, we summarized the phytochemical, pharmacological and pharmacokinetic investigations that have been conducted in recent years.

## Phytochemical investigation of HLJDD

The components of TCM formulas are complex, but not all of them have pharmacological activities. Therefore, it is of great significance to separate and identify such pharmacodynamic components. Many studies manifested that alkaloids from RC and CP, flavonoids from RS and terpenes from FG are three major active components in HLJDD and therefore are regarded as markers for quality control of HLJDD [35, 37–42]. In recent years, with the progress of modern detection technology, the majority of researchers have actively explored the chemical components in HLJDD and established qualitative and quantitative detection methods for some of its active components. By HPLC–UV/MS, 11 major peaks in the chromatogram of HLJDD extracted by water were identified as geniposide, jatrorrhizine, palmatine, berberine, baicalin, wogonoside, baicalein, wogonin, coptisine, oroxin A, obaculactone. Among them, coptisine and obaculactone were two characteristic peaks that could distinguish CR from CP. The following quantitative analysis showed that baicalin was the most abundant, followed by geniposide, then berberine and wogonoside, respectively [43]. However, the contents of berberine, baicalin, geniposide, and baicalein in HLJDD by decocting twice under refluxing with 70% ethanol (1:10 and then 1:5, w/v) were 5.12%, 4.17%, 1.65%, and 0.96%, respectively [44]. The reason for this difference may be related to different extraction methods and the conditions of HPLC. An effective quantitative method based on multiple wavelengths HPLC–DAD was developed for simultaneous determination of fourteen major ingredients (seven alkaloids, four flavonoids, three terpenes) in HLJDD. The total contents of these fourteen analytes reached to 70% [45]. With HPLC–UV analysis, the chemical profile of HLJDD samples was generated. HLJDD comprises four distinct constituents including berberine, palmatine, baicalin and geniposide in an approximate ratio of 3:1:1:3 [6]. Moreover, Q-Exactive was employed for the comprehensive chemical identification of HLJDD. 69 compounds, including alkaloids, flavonoids, iridoids, triterpenoid, monoterpene and phenolic acids were identified, 17 major characteristic constituents were selected as the quality control markers of HLJDD [46]. Currently, the analysis of active ingredients in HLJDD is focusing either on the prescription or on its extract, while quantification of that in biological samples have seldom been reported. A rapid and sensitive UHPLC-MS/MS method was developed to determinate seven main active constituents (berberine, palmatine, jatrorrhizine, baicalin, baicalein, wogonoside, and wogonin) simultaneously in atherosclerosis rat plasma after administration of HLJDD at doses of 1.5, 3, and 6 g/kg. Baicalin, baicalein, wogonoside, and wogonin were highly detected in a dose-dependent manner, while the other three components were determined in a quite low level and in a dose-independent mode [47]. In this review, the chemical components of the four herbs of HLJDD were summarized and classified, which will provide references for the separation and analysis of the chemical compositions of HLJDD.

### Alkaloids

**Fig. 2:**
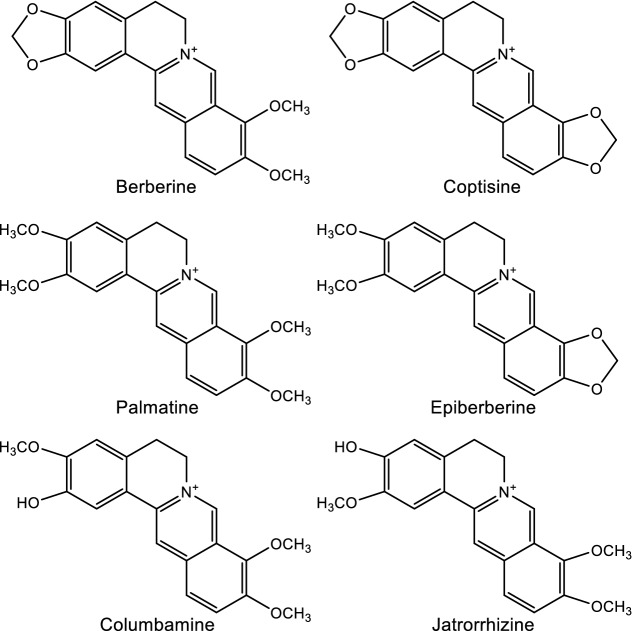
Structures of representative alkaloids isolated from HLJDD

Alkaloids are nitrogen-containing organic compounds responsible for the bitter taste of HLJDD. The main sources of alkaloids in HLJDD are RC and CP, which including protoberberine-type, oxyberberine-type, aporphine-type, indolopyridoquinazoline-type, and furoquinoline-type alkaloids. Berberine [48–55] and palmatine [49, 50, 52, 54–56] were isolated from *C. chinensis* Franch, *C. japonica* Makino, *P. chinense* Schneid and *P. amurense* Rupr. Coptisine [48–50, 52] and epiberberine [49, 50, 52, 56] were isolated from *C. chinensis* Franch. Columbamine [49, 50, 54, 56] and jatrorrhizine [49, 50, 54, 56] were isolated from *C. chinensis* Franch and *P. amurense* Rupr. The above six protoberberine-type alkaloids (Fig. 2) are considered as the main bioactive compounds of RC [57–65]. Moreover, the quantitative determination of berberine and palmatine is a very important index in the quality evaluation of CP.

The structural characteristics of alkaloids determine the low absorptions. Berberine, for example, is a quaternary ammonium alkaloid with conjugated double bonds and therefore has strong rigidity and poor solubility. Besides, berberine is the substrate of P-gp, which is an efflux transporter [47]. In addition, most berberine was excluded by the gastrointestinal tract after intragastric administration and was metabolized in a variety of pathways [66]. Hence, even long-term administration of alkaloids does not accumulate easily in the body because of their poor absorption through the intestinal wall.

### Flavonoids

**Fig. 3:**
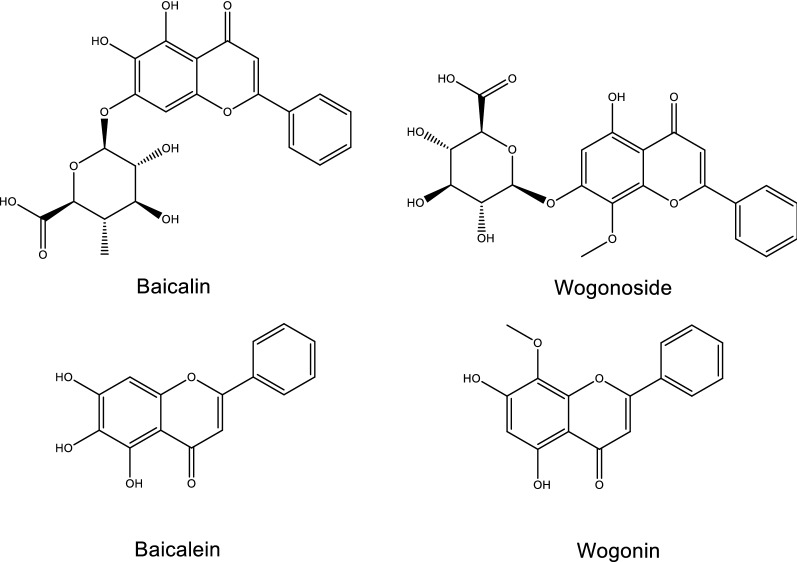
Structures of representative flavonoids isolated from HLJDD

Flavonoids, a class of polyphenol secondary metabolites, are broadly presented in plants and fungi. Their basic structure consists of C_6_–C_3_–C_6_ ring with different substitution patterns to produce a series of subclass compounds, including flavones, flavonols, chalcones, dihydrochalcones, aurones, flavanones, dihydroflavonols, and anthocyanins. Flavonoids are the most abundant and biologically active ingredients of RS, for more than 40 flavonoids have been discovered so far from RS in the form of aglycones and glycosides [67]. Among them, baicalin [68, 69], baicalein [68, 69], wogonoside [68, 69], and wogonin [68–70] (Fig. 3) are the characteristic chemical components of RS. By HPLC analysis, the ratio of the above four flavonoids in RS was 10.11%, 5.41%, 3.55% and 1.3%, respectively [71].

The absorptions of flavonoids were relatively better than the alkaloids. Flavonoids are easy to combine with glucuronic acid or sulfuric acid to form two-phase metabolism, thereby their plasma concentration–time curves showed obvious bimodal phenomena [72].

### Iridoids and iridoid glycosides

**Fig. 4:**
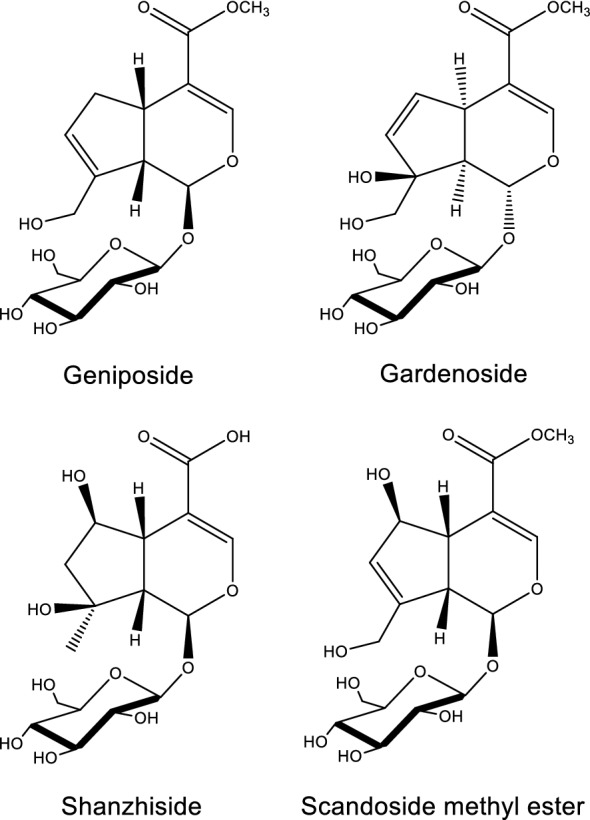
Structures of representative iridoid glycosides isolated from HLJDD

The major effective constituents of FG are iridoids and iridoid glycosides, such as genipin [73], geniposide [74–76], gardenoside [74, 76], shanzhiside [74, 76], and geniposidic acid [77] (Fig. 4). Among these components, geniposide and gardenoside, in particular, have very similar chemical compositions, with a difference of only one oxygen atom [77]. These compounds are responsible for the biological activities of FG, and their accurate and effective purification is of great significance for the quality control of this drug and its formulations. The content of iridoid glycosides may vary from different processing methods at about 2.65–7.23% [78]. A study quantified the content of geniposide, gardenoside, and geniposidic acid from different origins in China with 60.88 ± 11.47 mg/g, 56.33 ± 17.55 mg/g, and 2.61 ± 0.91 mg/g, respectively. Meanwhile, their average content were 52.80 ± 12.93 mg/g, 42.50 ± 13.21 mg/g, and 2.88 ± 2.19 mg/g, respectively, measured from different regions in Korea [77].

### Other chemical components

Lignans, polyphenolic substances derived from phenylalanine via dimerization of substituted cinnamic alcohols, are also abundant in RC. They have various structures, including benzofurans, furofurans, tetrahydrofurans, and arylnaphthanlenes [70, 79–81]. Moreover, phenylpropanoids [70, 80], phenolic compounds, saccharides, and steroids have been isolated from RC [50, 51, 80–83]. It was reported that diterpenoids [84, 85] and essential oils [86] were also found in RS. In addition to alkaloids, CP also contains sterols, esters, triterpenes and other compounds [87–90]. Triterpenes, monoterpenoids, carotenoids, phenolic acids and volatile ingredients were discovered in FG [91–93].

### Pharmacological effects

With the rapid development of modern pharmacology and biological technologies, increasing evidence has demonstrated the pleiotropic therapeutic functions of HLJDD on tumors, hepatic diseases, inflammations, allergies, blood lipid and glucose disorders, central nerve system diseases, bacterial infections, and intestinal flora disturbances (Table 1).

**Table 1:** Pharmacological actions and therapeutic or regulatory mechanisms of HLJDD

Pharmacological actions	Model	Mechanisms	Refs.
Anti-tumor	Hepatocellular carcinoma xenograft murine	Suppressing xenografted growth by inactivating eEF2 through the activation of AMPK signaling	[6]
	Hepatocellular carcinoma xenograft Hep G2 PLC/PRF/5	Inducing apoptosis Blocking cell cycle progression by regulating cell-cycle-related factor (p21/WAF1, cyclin B1, cyclin A, Cdc25C, and Cdc2) Promoting programmed cell death by modulating Bcl-2 Triggering mitochondrial pathway through membrane depolarization and caspase-9 activation Inhibiting NF-κB survival signaling pathway	[100]
Hepatoprotection	Thioacetamide	Restoring redox system, gut flora, and urea cycle	[24]
	Bile duct ligation	Restoring redox system, gut flora, Kreb’s cycle, and oxidation of branchedchain amino acids	[24]
	Bile duct ligation	Ameliorating energy metabolism, amino acid metabolism and gut microbiota metabolism Protecting oxidative injury	[25]
Anti-inflammatory	Carrageenan-induced rat air pouch A23187-stimulated peritoneal macrophages LPS-stimulated RAW 264.7 macrophages	Inhibiting inflammatory responses and eicosanoids generation from different lipoxygenases	[106]
	Carrageenan-induced mice paw edema LPS-stimulated RAW 264.7 macrophages	Reducing oxidative injury	[44]
	Collagen-induced arthritis rats	Regulating fatty acid oxidation and arachidonic acid metabolism	[19]
	LPS-stimulated RAW 264.7 macrophages	Suppressing the production of inflammatory mediators via inactivation of NF-κB and MAPKs, and degradation of IκBα	[108]
	Cecal ligation and puncture-induced septic model rats	Enhancing cholinergic anti-inflammatory pathway Inhibiting HMGB-1/TLR4/NF-κB signaling pathway	[4]
	Cecal ligation and puncture-induced septic model rats	Suppressing the production of proinflammatory cytokines Reversing the shift from Th1 to Th2 response and promote Th1/Th2 balance toward Th1 predominance Iinhibiting Th17 activation	[112]
	2,4-dinitrochlorobenzene-induced atopic dermatitis mice LPS-stimulated RAW 264.7 macrophages	Inhibiting MAPKs/NF-κB pathway	[115]
	LPS-induced gingivitis rats	Inhibiting AMPK and ERK1/2 pathway	[116]
	LPS-induced acute kidney injury mice	Inhibiting NF-κB and MAPK activation Activating Akt/HO-1 pathway Ameliorating disturbances in oxidative stress and energy metabolism	[26]
Anti-allergy	Antigen-induced RBL-2H3 cells	Suppressing allergic mediators via inactivation of MAPKs and Lyn pathway	[108]
Modulation of blood lipid	ApoE(-/-) mice Primary bone marrow-derived macrophage Foam cells	Regulating the functional differentiation of monocytes, macrophages, and foam cells	[119]
	High-fat diet-induced hyperlipidemia rats	Activating the activityof lipid metabolism enzyme Enhancing the expressions of LDLR and PPAR γ mRNAs	[14]
	High-fat diet and streptozotocin-induced T2DM rats	Inhibiting the activity of intestinal pancreatic lipase	[30]
Modulation of blood glucose	streptozotocin-induced T2DM rats	Enhancing GLP-1 secretion in gut to promoting insulin secretion and improving function of β cell	[120]
	Min6 cells NCI-H716 cells	Elevating intracellular cAMP levels to promote GLP-1 secretion and insulin secretion Increasing β cell mass through hyperplasia and hypertrophy	[121]
Central nervous system diseases	MCAO rats	Inhibiting neuron apoptosis and enhancing its proliferation through activating PI3K/Akt signaling pathway and HIF-1α	[28]
	MCAO rats	Inducing protective autophagy through the regulation of MAPK signals	[126]
	MCAO rats	Ameliorating the disordered metabolisms in energy, membrane and mitochondrial, amino acid and neurotransmitter Alleviating the inflammatory damage and the oxidative stress from ROS Recovering the destructed osmoregulation	[127]
	SAMP8	Modulating gene expressions in signal transduction (Dusp12, Rps6ka1, Rab26, Penk1, Nope, Leng8, Syde1, Phb, Def8, Ihpk1, Tac2, Pik3c2a), protein metabolism (Ttc3, Amfr, Prr6, Ube2d2), cell growth and development (Ngrn, Anln, Dip3b, Acrbp), nucleic acid metabolism (Fhit, Itm2c, Cstf2t, Ddx3x, Ercc5, Pcgfr6), energy metabolism (Stub1, Uqcr, Nsf), immune response (C1qb), regulation of transcription (D1ertd161e, Gcn5l2, Ssu72), transporter (Slc17a7, mt-Co1), nervous system development (Trim3), and neurogila cell differentiation (Tspan2)	[132]
	APPswe/PS1dE9 mice	Ameliorating neuroinflammation and sphingolipid metabolic disorder	[34]
	HEK 293 cells	Inhibiting indoleamine 2,3-dioxygenase activity	[133]
Anti-infection	*Candida albicans*	Inhibiting formation of hyphae and colony morphologies through downregulating the expression of HWP1, ALS3, UME6 and CSH1	[136]
	*Pseudomonas aeruginosa*	Reducing pyocyanin pigment, elastolytic activity, proteolytic activity, biofilm formation, and bacterial motility	[137]
	H1N1	Inhibiting NA activity	[139]
Modulation of microbiota	High-fat diet and streptozotocin-induced T2DM rats	Ameliorating hyperglycemia and restoring the disturbed gut microbiota structure and function through increasing short chain fatty acids-producing bacteria while reducing conditioned pathogenic bacteria	[143]

### Anti-tumor

The ancient Chinese medical monograph “Zhong-Zang-Jing” recorded some descriptions of cancer-like symptoms such as “Yong, Yang, Chuang and Zhong”, which are caused by retention of various pathogens including heat and dampness. Tumor growth involves induction of cell-cycle progression, avoidance of apoptosis, and activation of the cell survival pathway [94]. Modern studies indicated that HLJDD could disrupt these processes, to suppress the tumor growth in vivo, and inhibite proliferation of cancer cells in vitro.

**Fig. 5:**
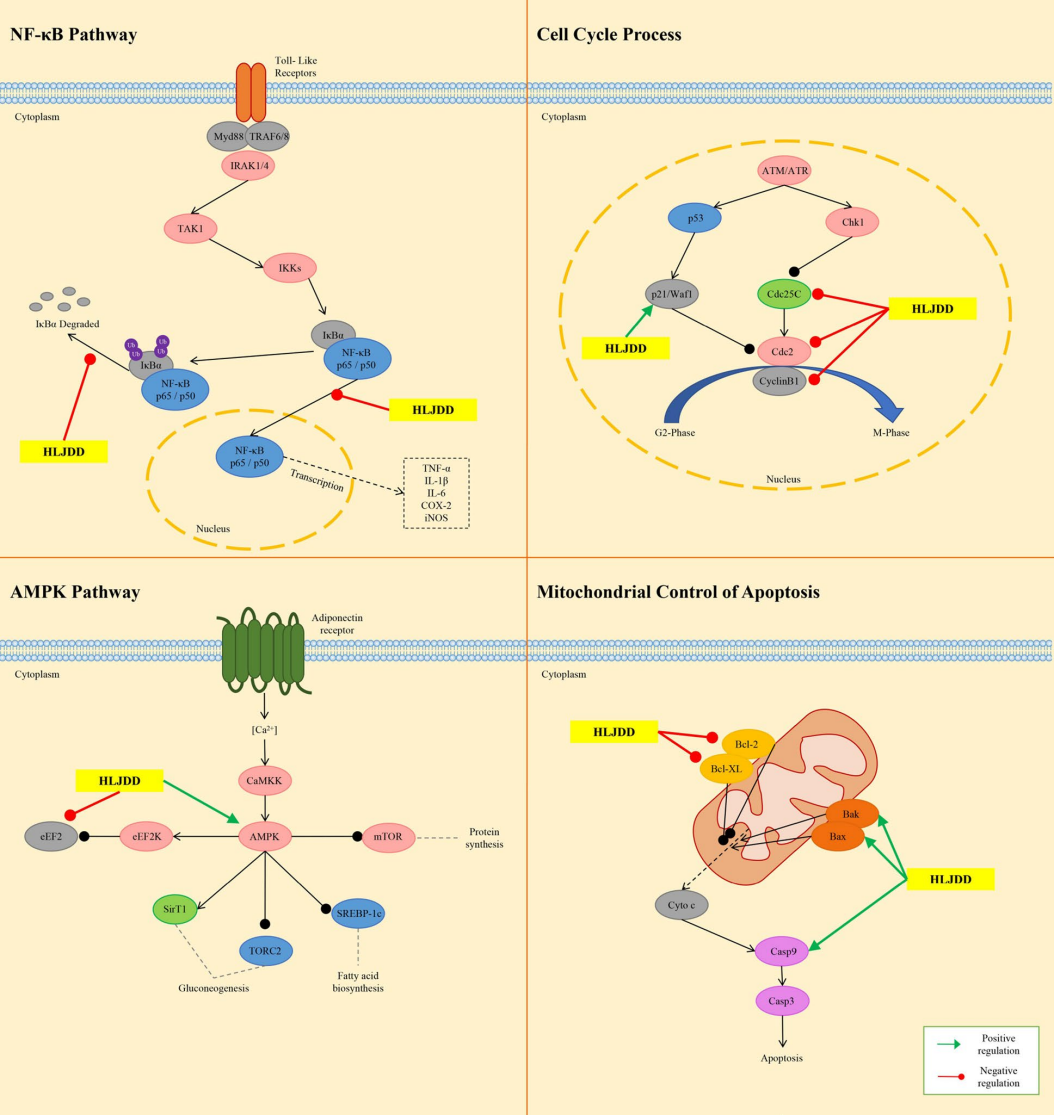
Molecular mechanisms of anti-tumor effect of HLJDD

In a hepatocellular carcinoma xenograft murine model, HLJDD was shown to suppress the xenografted growth in dose-dependent manner. The inhibitory effect of HLJDD may be due to the activation of eukaryotic elongation factor-2 kinase (eEF2K) and inactivation of eEF2. The activation of AMP-activated protein kinase (AMPK) signaling may be responsible for the eEF2K induction [6]. eEF2 is an essential protein for the elongation of nascent peptide [95]. The inactivation of eEF2 suppresses the synthesis of nascent protein, which supports the proliferation of the cancer cells [96]. The AMPK activation was reported to inhibit the mammalian target of rapamycin (mTOR) activity, followed by blockade of mTOR-mediated eEF2K phosphorylation [97]. Geniposide, baicalin, berberine and palmatine could induce phosphorylated eEF2 expression in Hep G2 and MHCC97L cells, which suggested that these four compounds could target on eEF2 [6]. However, their inhibitory effects on eEF2 activity have not been reported. Berberine and baicalin may be the two main components targeting AMPK in HLJDD, since they have been reported as AMPK activators [98, 99]. It would be quite interesting to investigate the precise mechanism of the combination effect of active compounds in HLJDD. Another study on HLJDD in the treatment of hepatocellular carcinoma revealed the mutiple underlying mechanisms, including induction of apoptosis, blockade of cell cycle progression by regulating cell-cycle-related factor, modulation of the B cell CLL/lymphoma 2 family proteins to favor programmed cell death, triggering of the mitochondrial pathway through membrane depolarization and caspase-9 activation, and inhibition of nuclear factor-kappa B (NF-κB) survival signaling pathway [100]. RS was responsible for the suppressive effect of HLJDD on myeloma cell proliferation, since RS alone exhibited stronger growth inhibition (IC_50_ 30 ng/mL) than HLJDD (IC_50_ 70 ng/mL) on U266 cells. In addition, baicalein showed the strongest growth inhibition with an IC_50_ of 28 μM; while the IC_50_s of baicalin and wogonin, another two major flavonoids of RS, were greater than 200 μM. Baicalein inhibited the survival of MPC-1^−^ immature myeloma cells in vitro, and induced apoptosis in myeloma cell lines by inhibiting the activity of NF-κB and thereby blocking the degradation of inhibitor-kappa B-alpha (IκB-α). Further, induction of apoptosis by HLJDD, RS or baicalein may be considered to be involved in the mitochondria-mediated pathway, because the rapid loss of mitochondrial membrane potential was confirmed, followed by enhanced release of cytochromes *c* and subsequent activation of caspase-9 and caspase-3 [101]. In mitochondria pathway, the activity of NF-κB is considered to be pivotal, which modulates the expression and function of B-cell CLL/lymphoma 2 family proteins in the mitochondria [102, 103]. These findings, consistent with previous studies, suggested that HLJDD and its active components exert therapeutic effects on different tumors through almost the same pathway. The molecular mechanisms of HJDD against tumor are shown in Fig. 5.

The role of TCM in the treatment of tumors is often auxiliary. Combined with chemical drugs, it can increase the efficacy on one hand, and reduce the side effects on the other hand, while the researches in these aspects need to be further studied.

### Hepatoprotection

Liver is vital for bile formation, amino acid utilization and ammonia detoxification, and is also the organ where glycolysis, gluconeogenesis, and the synthesis of certain plasma proteins happen [104]. In the liver, the toxic chemicals are commonly metabolized by cytochrome P450, namely first-pass effect. Hence, its detoxification ability would be attenuated due to pathological damage. In TCM, liver is depicted as an organ susceptible to heat and toxins, due to which dysfunction of liver is observed.

HLJDD is rich in bioactive alkaloids, flavonoids, iridoid glycosides, and polyphenols, could restore the balance of the disturbed metabolic status common in two cholestasis injuries, e.g. redox system and gut flora, urea cycle in thioacetamide model, and Kreb’s cycle and oxidation of branchedchain amino acids in bile duct ligation model, respectively [24]. These findings are consistent with a previous study that also used bile duct ligation to induce cholestatic liver injury [25]. The protection of single or combination use of berberine and HLJDD on acute liver injury induced via cecal ligation and puncture were to explore the herb-drug interactions of them in a holistic way. Livers of sham-operated group, treatment groups of berberine, HLJDD and their co-administration displayed no obvious histopathological changes. Both histamine and trimethylamine *N*-oxide were exclusively decreased by the treatments of HLJDD with or without berberine. Glutathione and carnosine were significantly increased after HLJDD and the combination treatment. Metabolomics analysis revealed that HLJDD had better anti-inflammatory, anti-bacterial, and anti-oxidative effects than berberine alone. The single use of berberine had an inferior ability to HLJDD in restoring the whole disturbed metabolism of model rats [105].

### Anti-inflammatory and anti-allergy

It is believed in TCM that endogenous and exogenous heat and toxins are pathogenic mechanisms of inflammation. To some extent, inflammatory and allergic mediators, as well as inflammatory factors generated by inflammations and allergies are recongnized as toxins leading to the heat syndromes appearing in the context of inflammatory and allergic responses.

Oral administration of HLJDD at a dose of 150 mg/kg and 300 mg/kg significantly inhibited the inflammatory responses in carrageenan injected rat air pouches, with the inhibition ratio for exudate volume being 22.1% and 25.7%, and for leucocyte influx 26.4% and 36.2%, respectively. It also greatly reduced the production of nitric oxide (NO) and leukotriene B (4) in vivo without any influence on the biosynthesis of cyclooxygenase-derived eicosanoids. However, eicosanoids derived from different lipoxygenases (LOs) were markedly inhibited by HLJDD in calcium ionophore A23187-stimulated peritoneal macrophages [106]. Further experiments on cell-free purified enzymes showed that RC and RS were responsible for the suppressive effect of HLJDD on eicosanoid generation. Baicalein and baicalin derived from RS showed significant inhibition on 5-LO and 15-LO, and coptisine derived from RC showed medium inhibition on leukotriene A_4_ hydrolase. Moreover, 6 pure components including baicalein, baicalin, wogonoside, wogonin, coptisine, and magnoflorine could inhibite the generation of eicosanoid in rat peritoneal macrophages via LO pathway [11]. In lipopolysaccharide (LPS)-treated RAW 264.7 macrophages, the NO production [44, 106], the mRNA expression of inducible nitric oxide synthase and several chemotactic factors (CCL3, CCL4, CCL5 and CXCL2) were suppressed by HLJDD [106]. Moreover, HLJDD also decreased the levels of malondialdehyde, prostaglandin E2, interleukin-6 (IL-6), IL-10, and tumor necrosis factor-alpha (TNF-α), and increased the activity of superoxide dismutase in this model [44]. The results of exploring the material base for the anti-inflammatory activity of HLJDD showed that its two fractions had different effects on these parameters. On one side, HLJDD-1 (iridoids and flavonoid glycosides) showed higher antioxidant activity than HLJDD-2 (alkaloids and flavonoid aglycones) as supported by decreasing the level of malondialdehyde and enhancing the activity of superoxide dismutase. On the other side, HLJDD-2 has a more obvious inhibitory effect on NO and IL-6 than HLJDD-1. Moreover, most of the four typical compounds (geniposide, baicalin, berberine and baicalein) of HLJDD showed weaker effects on these parameters than HLJDD and the two fractions, suggesting that these compounds may have synergistic anti-inflammatory interactions [44]. In collagen-induced arthritis rats, the combination of 13 components (geniposide, coptisine, phellodendrine, jatrorrhizine, magnoflorine, palmatine, berberine, baicalin, chlorogenic acid, crocin, wogonoside, baicalein, and wogonin) of HLJDD exhibited similar pharmacological activities as HLJDD aqueous extracts in ameliorating the symptoms of arthritis, preventing joint damage, and reducing the serum levels of TNF-α, interferon-gamma and IL-17 [107]. HLJDD and its constituents combination have been shown to regulate fatty acid oxidation and arachidonic acid metabolism in collagen-induced arthritis rats [19]. In addition, the disturbed urinary levels of succinic acid, citric acid, creatine, uridine, pantothenic acid, carnitine, phenylacetylglycine, allantoin and plasma levels of phenylpyruvic acid in model rats were demonstrated to be restored by HLJDD. Meanwhile, the combination of HLJDD was able to recover the disordered urinary levels of citric acid, creatine, pantothenic acid, carnitine, phenylacetylglycine and plasma levels of uric acid, l-histidine, and l-phenylalanine in model rats [17]. Taken together, the 13 constituents’ combination may represent the effective-composite of HLJDD. More importantly, HLJDD is beneficial in suppressing inflammation processes by synergistically acting on various components that on multiply target point. Hence, further researches elucidating the mode of action of these ingredients would give an insight into the use of HLJDD for its anti-inflammatory activity. The results of in vitro experiments indicated that the ethanolic extract of HLJDD exerted significant anti-inflammatory and anti-allergic effects through suppressing the production of inflammatory mediators (NO, IL-1β, IL-4, monocyte chemoattractant protein-1and granulocyte–macrophage colony stimulating factor) via the NF-κB and mitogen-activated protein kinases (MAPKs) inactivation and IκB-α degradation in the LPS-stimulated RAW 264.7 cells, and allergic mediators (IL-4, TNF-α, and monocyte chemoattractant protein-1) by inactivating the MAPKs and Lyn pathway in antigen-induced RBL-2H3 cells [108].

**Fig. 6:**
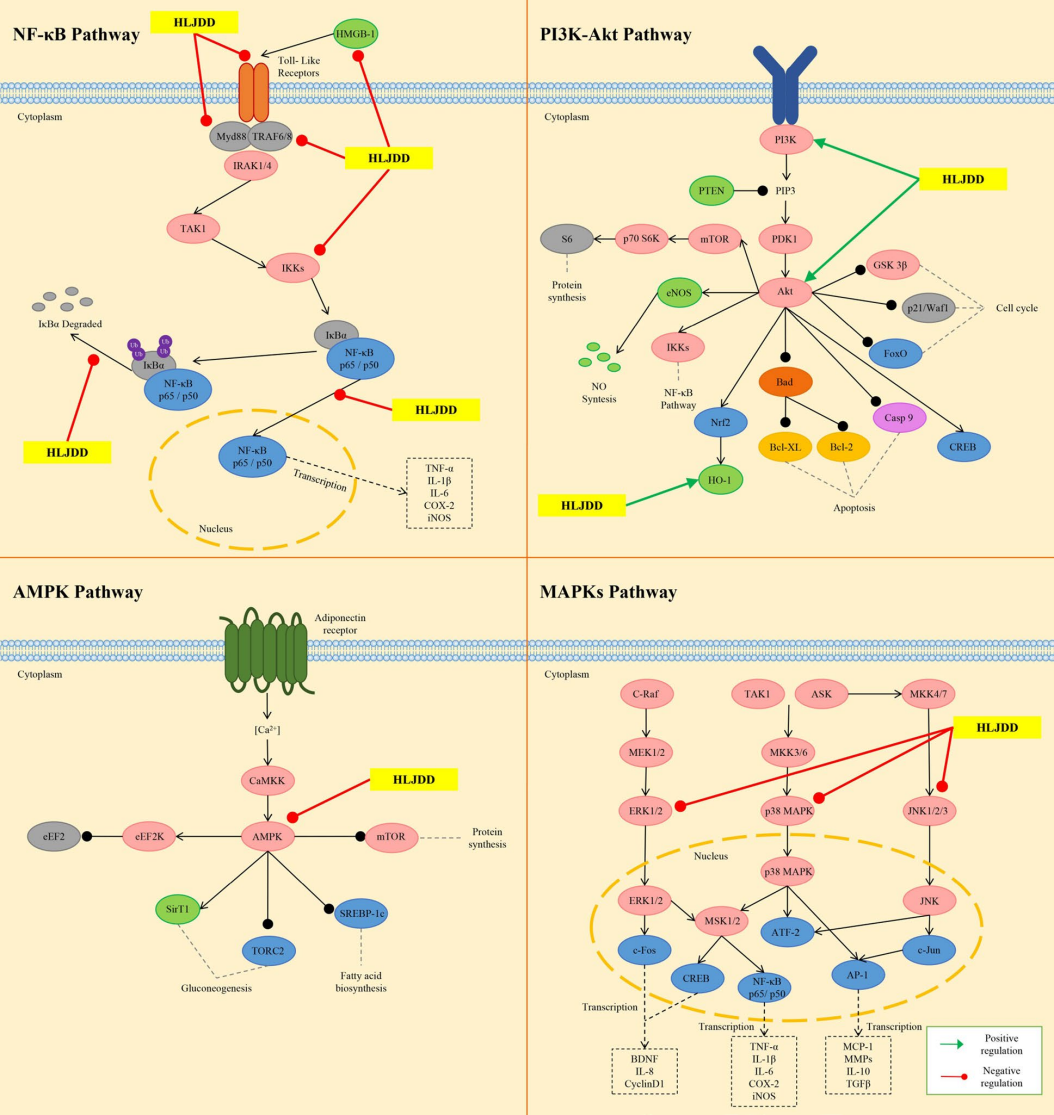
Molecular mechanisms of anti-inflammatory effect of HLJDD

Based on its powerful anti-inflammatory ability, a large number of studies have proved that HLJDD is an effective prescription for treating various inflammatory diseases, such as inflammatory bowel disease [109], gastritis [110, 111], and sepsis [4, 22, 112–114]. Sepsis is a clinical syndrome characterized by systemic inflammation. In the experimental septic model rats induced by cecal ligation and puncture, HLJDD treatment suppressed the production of proinflammatory cytokines (TNF-α, IL-1, IL-6, and IL-17A), reversed the shift from T-helper (Th) 1 to Th2 response and promote Th1/Th2 balance toward Th1 predominance, and inhibited Th17 activation [112]. In addition, l-proline, l-valine, oleic acid, carnitine, palmitoylcarnitine, arachidonic acid, and arachidic acid were reversed by HLJDD, while docosahexaenoic acid, eicosapntemacnioc acid, and prostaglandin E3 were further elevated by HLJDD in the septic condition [22]. The strong therapeutic effects of HLJDD in septic models may be ascribed to its significant enhancement of cholinergic anti-inflammatory pathway and inhibition of high mobility group protein B1/Toll-likereceptor 4/NF-κB signaling pathway [4]. Sepsis often result in endorgan dysfunction, such as acute kidney injury. HLJDD and its component herbs could effectively inhibit LPS-induced acute kidney injury in mice by inhibiting NF-κB and MAPK activation and activating the Akt/HO-1 pathway, and by significantly ameliorating disturbances in oxidative stress and energy metabolism induced by LPS [26]. At present, in vivo and in vitro studies also indicated that HLJDD showed atopic dermatitis treatment effects. In 2,4-dinitrochlorobenzene-induced atopic dermatitis mice, HLJDD down-regulated serum expression levels of IL-1α, IL-1β, IL-2, IL-4, IL-5, IL-6, interferon-gamma and TNF-α, normalised the splenic CD4^+^/CD8^+^ T-lymphocyte ratio, and inactivated MAPKs (including p38, extracellular regulated protein kinases (ERK), and c-Jun N-terminal kinase (JNK)), IκB-α, and NF-κB (p65). Moreover, HLJDD inhibited LPS-induced differentiation of RAW264.7 cells, reduced LPS binding to the RAW264.7 cell membrane, as well as decreased ERK, p38, JNK, IκB-α, and p65 phosphorylation levels in the MAPKs/NF-κB pathway and inhibited p65 nuclear translocation [115]. Further, a study revealed that HLJDD had a positive effect in rat gingivitis induced by LPS. HLJDD boosted the ability of anti-oxidation and anti-inflammatory by inhibiting AMPK and ERK pathways [116]. The molecular mechanisms of HLJDD regulating inflammation-related pathways are shown in Fig. 6.

According to the “Four-nature Theory”, all Chinese herbs are fit into four categories, including “cold” “hot” “warm” and “cool” herbs. Based on this theory, the four herbs in HLJDD are all recognized as “heat-clearing” herbs, which means that they all have therapeutic powers of removing the “body fire”. In classic Chinese philosophy, “fire”, one of five “basic elements” (wood, fire, earth, metal and water), is an element with dual seemingly paradox roles as both beneficial and deleterious [117]. The excess “body fire” will exert deleterious impacts and form the basis of many diseases. In fact, the essence of “body fire” is a gradual process including oxidative/nitrosative stress, inflammation and infection. Oxidative stress can induce inflammation and many other diseases by disrupting normal cellular mechanisms. Infection is a form of invasion and multiplication of various infectious agents in body, which will also cause inflammation. Therefore, inflammation involves a greatly complex web of intercellular cytokine signals [118] and is related to the pathogenesis of most diseases, such as cancer, and CNS diseases mentioned in this review.

### Blood lipid and glucose-modulating

Symptom-complex of *wasting*-*thirst* in TCM mainly refers to syndrome X. According to the classical TCM theory, the pathogenesis of the metabolic syndrome is induced by excessive “heat” dissipating the body fluids. Moreover, recent researches on TCM theory pointed out that the internal heat is the primary pathogenic factor for the development of T2DM. In addition, excessive lipid may lead to the accumulation of “heat”, which eventually transforms into toxin, a more serious cause.

The regulations of lipid are divided into several parts: reducing lipid synthesis; increasing lipid degradation; and combating damage caused by high levels of lipids, such as inducing inflammatory responses. In an apolipoprotein E knockout mouse model, HLJDD was found to markedly decrease the ratio of inflammatory subset of monocytes. In addition, the results from in vitro experiments indicated that HLJDD-containing serum significantly facilitated differentiation of M2 macrophages and foam cells. Thus, HLJDD might attenuate the development of atherosclerosis, probably by regulating the functional differentiation of monocytes, macrophages, and foam cells [119]. It was reported that HLJDD could activate the activity of lipoprotein lipase and hepatic lipase, and enhance the expressions of low-density lipoprotein receptor and peroxisome proliferator-activated receptor gamma mRNAs to modulate the lipid metabolism in high-fat diet-induced rats [14]. However, HLJDD contains various chemical components and might possess multiple mechanisms to modulate the lipid metabolism. Therefore, HLJDD may exert the hypolipidemic effect through other mechanisms. For example, using the olive oil loading test, Zhang et al. reported that HLJDD extract lowered total cholesterol, triglyceride, and low-density lipoprotein cholesterol level of T2DM rats by inhibiting intestinal pancreatic lipase activity [30]. It could be speculated that HLJDD might exert the effect of lipid-modulating by multi-targets, multi-pathways and multi-effects.

Insulin secretion and insulin action are essential for blood glucose homeostasis, and defects in either process cause metabolic diseases, such as T2DM [120, 121]. Furthermore, HLJDD could decrease blood glucose concentration and ameliorated diabetic syndrome partly through its interaction with intestinal tract [120]. Glucagon-like peptide 1 (GLP-1), an important incretin secreted by the gastrointestinal L-cells, enhances insulin secretion, improves β cell proliferation and neogenesis, and reduces glucagon release from the pancreatic islet cells [122, 123]. In the last decade, a novel group of glucose-lowering agents has been developed based on the gut hormone GLP-1 [124]. It was reported that 5-week HLJDD (4 g/kg/day) treatment on diabetic rats enhanced GLP-1 secretion in gut and the released GLP-1 subsequently promoted insulin secretion and improved function of β cell in pancreas [120]. In an in vitro study, the water extracts of RS and HLJDD increased insulin secretion in Min6 cells and GLP-1 secretion in NCI-H716 cells by elevating intracellular cyclic adenosine monophosphate levels. RS and HLJDD also increased β cell mass through hyperplasia and hypertrophy. The rise in hyperplasia was associated with elevated insulin receptor substrate 2 and pancreatic and duodenal homeobox 1 expression in the islets [121]. Geniposide, an active ingredient of HLJDD, has been reported as an agonist for GLP-1 receptor [125]. However, whether it can promote GLP-1 secretion is still unclear. Moreover, whether other compounds included in HLJDD contribute to the promotion of GLP-1 remains to be further investigated.

### Central nervous system diseases

Diseases of central nervous system (CNS) are also believed to have close associations with the heat and toxins in TCM theory. The pathogenic factors, namely toxins, lead to nervous system injury, both in function and/or organic architecture. The typical clinical symptoms are dysfunction of learning and memory, mood disorder, psychosis, cerebrovascular diseases, etc.

Currently, considerable studies have been conducted to understand the pharmacological mechanisms of HLJDD on ischemia-induced brain damage. Preconditioning of HLJDD protected neurons against oxygen and glucose deprivation, significantly reduced the cerebral infarction volume and cerebral water content, and improved the neurological deficient score of model rats obtained through middle cerebral artery occlusion (MCAO). The activation of the phosphatidylinositol 3-kinase/protein kinase B (Akt) signaling pathway and hypoxia-inducible factor-1 alpha was proved to be responsible for the resistance of HLJDD to ischemia–reperfusion or hypoxia injury contribute to inhibiting neuron apoptosis and enhancing neuron proliferation [28]. Furthermore, it has been reported that HLJDD exerted neuroprotective effects on ischemic stroke partly though the Akt-independent protective autophagy via the regulation of MAPK signals, which can avoid unfavorable side-effects associated with the inactivation of Akt [126]. Pattern analysis of the ^1^H NMR data disclosed that HLJDD could relieve MCAO rats by ameliorating the disordered metabolisms in energy, membrane and mitochondrial, amino acid and neurotransmitter, alleviating the inflammatory damage and the oxidative stress from reactive oxygen species, and recovering the destructed osmoregulation [127]. Total alkaloids, iridoids and flavonoids from HLJDD have potential as a treatment for ischemic brain injury. Firstly, alkaloids treatment was found to enhance neurogenesis by increasing the expression of vascular endothelial growth factor, angiopoietin-1 (Ang-1), and Ang-2 protein, and its neuroproliferative effect was partially correlated with enhanced phosphorylation of Akt, and glycogen synthase kinase-3 beta. Secondly, flavonoids could promote differentiation of cortical precursor cells into neuronal, which may be attributable to the regulation of Akt, glycogen synthase kinase-3 beta mRNA and Ang-1 protein levels. Finally, alkaloids and iridoids increased number of BrdU-positive cells and enhanced neuronal differentiation in the cortex [29]. Berberine, baicalin and gardenoside are the representative components of alkaloids, flavonoids and iridoids respectively, all of which can improve functional outcome after brain ischemia. Berberine exerted potent neuroprotective effects in ischemic environment [128]. Baicalin could also protect neuronal cells against various neurotoxic stimuli and ischemia–reperfusion injury [40]. Gardenoside was shown to enhance neurons viability, prompt neurite growth, and attenuate neuronal death against ischemic damage [129]. A study showed that the combination of these three ingredients treatment increased the levels of cellular antioxidants that scavenged reactive oxygen species during ischemia–reperfusion via the nuclear erythroid 2-related factor 2 signaling cascade, and exhibited stronger effects than the individual herbs alone [130]. Berberine and baicalin were the molecular basis for ameliorating the neurological function in ischemia–reperfusion, possibly due to their induction of increased expression of NF-κB, inducible nitric oxide synthase and cyclooxygenase 2 protein. In addition, the combination of berberine and gardenoside possessed neuroprotective effects, which may be related to their regulation of oxidative stress and autophagy [131]. These results indicated that the synergistic effects of different components of HLJDD are responsible for the powerful effectiveness of HLJDD. Besides, HLJDD was proved to ameliorate neurodegenerative diseases, such as AD. Clinical signs of AD are characterized by the neuron loss and cognitive impairment. Modern pharmacological studies have showed that HLJDD could significantly modulate effects on age-related changes of the gene expressions in the hippocampus and cerebral cortex in SAMP8 model, which include genes that involved in different biological function and process: signal transduction (Dusp12, Rps6ka1, Rab26, Penk1, Nope, Leng8, Syde1, Phb, Def8, Ihpk1, Tac2, Pik3c2a), protein metabolism (Ttc3, Amfr, Prr6, Ube2d2), cell growth and development (Ngrn, Anln, Dip3b, Acrbp), nucleic acid metabolism (Fhit, Itm2c, Cstf2t, Ddx3x, Ercc5, Pcgfr6), energy metabolism (Stub1, Uqcr, Nsf), immune response (C1qb), regulation of transcription (D1ertd161e, Gcn5l2, Ssu72), transporter (Slc17a7, mt-Co1), nervous system development (Trim3), and neurogila cell differentiation (Tspan2) [132]. In APPswe/PS1dE9 mice, another classic animal model of AD, HLJDD had positive effects on AD by ameliorating neuroinflammation and sphingolipid metabolic disorder [34]. In addition, HLJDD may inhibit the activity of indoleamine 2,3-dioxygenase, one of the potential participants involved in the pathogenesis of AD [133].

**Fig. 7:**
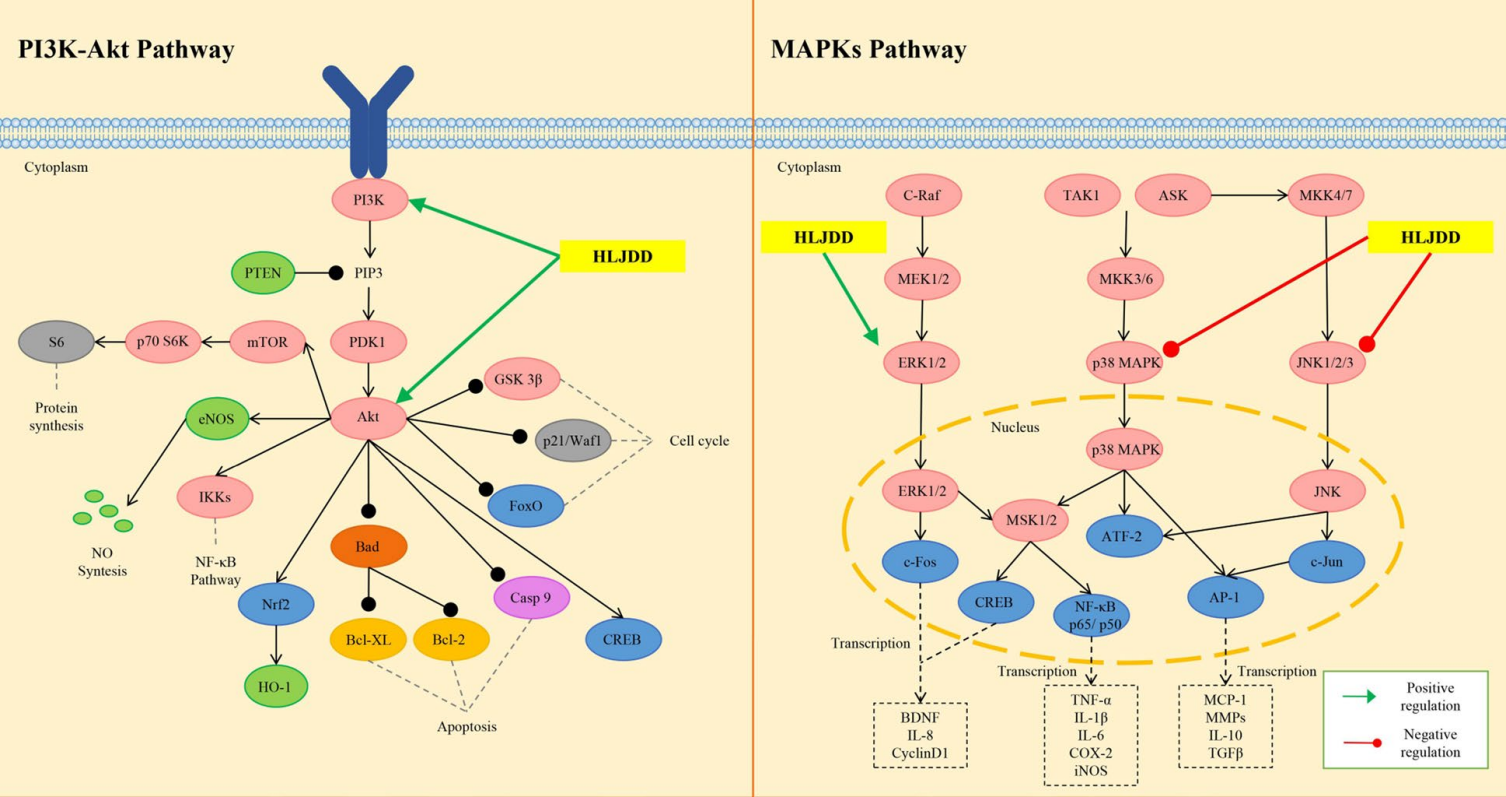
Molecular mechanism of HHLJDD in treating CNS diseases

The effects of HLJDD on CNS diseases are mainly through anti-inflammatory, antioxidant, and regulating energy metabolisms. At the same time, HLJDD also has different effects on central nervous functions and neurotransmitter levels. In a metabolomics study, HLJDD decreased the levels of glutamine and γ-aminobutyric acid in plasma of MACO rats, which might be responsible for neuronprotection via the decline of excitotoxicity of glutamate. HLJDD also elevated acetylcholine level and maintained cholinergic neurons function [27]. The molecular mechanisms of HLJDD in the treatment of CNS are shown in Fig. 7.

However, the role of HLJDD in the CNS should not be considered simply from the traditional pharmacological effects. New perspectives, such as regulating the liver and gut bacteria, should be given more attention. The former can regulate the CNS through the liver–brain axis, while the latter can further intervene the CNS by activating the brain–gut axis, especially in the case of mental system diseases. The inflammatory response of the CNS is an important link and target for the intervention of HLJDD in the CNS. Compounds that directly enter the brain tissue, as well as the liver–brain axis and the brain–gut axis, are the pathways for the effects of HLJDD. The regulation of energy metabolism, on one hand, has a direct antagonistic effect on the occurrence and development of cerebrovascular diseases. On the other hand, the adjustment of energy can also adjust the function and state of microglia to intervene inflammation.

### Anti-infection and microbiota-modulating

Bacterial or virus infection or imbalance of bacteria in the body commonly stimulates inflammatory responses or immune activation resulting in redness, swelling, heat and pain directly or participates in the pathological development of various systems such as gastrointestinal tract, endocrine and CNS. Such diseases are covered, at least partly, by the theory of heat and toxins of TCM. The actions on bacteria or viruses are the focus of heat dissipation and detoxification treatments.

*Candida albicans* (*C. albicans*) is the most prevalent opportunistic fungal pathogen that can cause surface and even systemic infections in immunocompromised patients [134, 135]. The results of gene expression of *C. albicans* with the treatment of HLJDD showed that ATP-binding cassette transporter and major facilitator superfamily transporter, which encode multidrug transporters, were identified to be remarkably upregulated, which might provide insights for the inhibition mechanism of HLJDD against *C. albicans* [35]. The ethyl acetate extract of HLJDD with concentration of 312 mg/L and 1250 mg/L could inhibit formation of hyphae and colony morphologies of *C. albicans* through downregulating the expression of hyphae-specific genes such as HWP1, ALS3, UME6 and CSH1 [136]. *Pseudomonas aeruginosa*, an opportunistic Gram-negative pathogen, has characteristic of quorum sensing modulation. HLJDD showed the lowest minimum inhibitory concentration (MIC) of 100 mg/mL against *Pseudomonas aeruginosa*, while MICs of 200 mg/mL for the RC and RS, 400 mg/mL for the CP, and more than 400 mg/mL for the FG. Moreover, at the sub-MIC, HLJDD significantly reduced pyocyanin pigment, elastolytic activity, proteolytic activity, biofilm formation, and bacterial motility [137]. In *Mugil cephalus*, 1% modified HLJDD feeding for 28 days may prevent *Lactococcus garvieae* infection and could be used in aquaculture industries [138]. Moreover, the water extracts of HLJDD and its four herbs exerted potent treatment power on H1N1 infection through the inhibition of neuraminidase (NA) activity [139], which is one of the biomarkers for subtype classification of influenza A virus. The IC_50_ of HLJDD, RC, RS, CP, FG, and peramivir (positive control) on NA activity were 112.6 ± 6.7 μg/mL 96.1 ± 7.6 μg/mL, 303.5 ± 21.9 μg/mL, 108.6 ± 8.6 μg/mL, 285.0 ± 16.6 μg/mL, and 478.8 ± 15.6 μg/mL, respectively. Accordingly, it is valuable to use HLJDD as a complementary medicine for H1N1 infection in clinical. In addition, based on the effective inhibitors of various NA subtypes of its active ingredients, such as berberine [140], coptisine [141], and baicalein [142], it is meaningful to further study the anti-viral effect of HLJDD.

In high-fat diet and streptozotocin-induced T2DM rats, HLJDD treatment ameliorated hyperglycemia and restored the disturbed gut microbiota structure and function to a nearly normal condition mainly through increasing short chain fatty acids-producing bacteria while reducing conditioned pathogenic bacteria [143].

Various chemical components in HLJDD have anti-infection effects, especially alkaloids, of which berberine has been used as a commodity for the treatment of bacterial diarrhea. For bacteria of different species, there are commonalities and differences between different components. At present, researches on bacteria cannot be limited to bacteriostatic or bactericidal. In view of the low bioavailability of chemical components, the effects of intestinal flora on the metabolism of compounds in HLJDD, as well as the evaluations of level and activity of metabolites need to be further studied. In a metabolomics study, 6 high level compounds in HLJDD [46], including 4 alkaloids (berberine, palmatine, coptisine and jatrorrhizine), 1 flavonoid (baicalin) and 1 iridoid (geniposide), were selected to clarify the metabolic pathways of HLJDD in rat urine and feces by LC–IT-MS combining with LC–FT-ICR-MS. In general, phase I (hydroxylation and demethylation) and phase II (sulfate conjugation and glucuronidated conjugation) reactions of flavonoids and iridoids, as well as phase I and II (hydroxylation, demethylation and glucuronidation) reactions of alkaloids were observed as the major metabolic fate of HLJDD in vivo. Notably, abundant benzylisoquinoline alkaloids were detected in feces due to their poor absorption in gastrointestinal tract. All the glucuronidated flavonoid glycosides were prototypes as well as metabolites [144]. It was reported that hydrolyzation by enterobacteria and subsequently glucuronidation reactions of flavonoids occurred in vivo [145]. In addition, the studies on the effects of the chemical components in HLJDD on the species abundance and metabolic activities of intestinal bacteria and the level of metabolites, such as neurotransmitters and short-chain fatty acids, may be new research ideas and directions to reveal the potential mechanisms and pathways of HLJDD.

### Other pharmacological effects

Early studies showed that HLJDD could protect ethanol- and aspirin-induced gastric mucosal barrier injury [146], and gastric hemorrhagic lesions [147]. These gastric protection effects of HLJDD may be ascribed to the reinforcement of mucosal barrier resistance through endogenous sulfhydryl compounds and diethyldithiocarbamate-sensitive compounds [148, 149]. In addition, HLJDD could inhibit drug-stimulated gastric acid secretion [150] via dopamine receptors and alpha-2 adrenoceptors [151]. In clinical, modified HLJDD combined electroacupuncture could promote the recovery of gastrointestinal function in critically ill patients after abdominal surgery via improving intestinal barrier function [152]. In addition, the administration of HLJDD in combination with chlorpromazine would alleviated the side-effects caused by less dose [153], while the mechanism remains to be unknown.

## Pharmacokinetic investigation

PK is a discipline which studies quantitatively the law of absorption, distribution, metabolism and excretion of drugs in vivo and expounds the law of blood drug concentration with time by applying mathematical principles and methods. PK investigation is of great significance in the new drug development, the studies of drug-induced toxicity, and drug interaction [154, 155]. Reasonably, it is the pivotal approach to reveal the obscure pharmacodynamic properties and toxicity of herbals or formulas in TCM [156]. Commonly, LC–MS/MS [157], HPLC–MS/MS [158], UPLC–MS/MS [159], and GC–MS [160] are the main techniques employed in PK investigation.

HLJDD is a traditional Chinese prescription with different types of PK interactions among its multi-components. In recent years, studies of the PK profiles and absorption of alkaloids, flavonoids and iridoid glycosides both in pure components and in HLJDD have been well conducted [38–41, 161–166], especially of berberine, baicalin and geniposide. Berberine had better absorption within HLJDD than that of solo compound in an intestinal perfusion model of rat [167]. Similar phenomena were observed in the study of investigating the differences of absorption of geniposide after oral administration of geniposide alone and HLJDD by PK studies in vivo, intestinal perfusion model, and Caco-2 model. In addition, geniposide had better absorption in the duodenum and jejunum through passive diffusion [168]. These results indicated that the intestinal absorption of berberine and geniposide were affected by compatibility of other compounds of HLJDD. Baicalin showed bimodal phenomenon in the plasma following oral administrations of pure baicalin and HLJDD in rats, and other components in HLJDD had PK interaction with baicalin [40]. There were few studies on the PK investigation of the whole HLJDD extracts. Ren et al. obtained systematic PK data concerning the activity of HLJDD under inflammatory conditions by LC-QqQ-MS using a dynamic multiple reaction monitoring method. In normal group, the C_max_ of geniposide, magnolflorine, baicalin, berberine, oroxylin A-7-*O*-glucuronide, wogonoside, wogonin and oroxylin A were 0.7 ± 0.3, 0.6 ± 0.2, 0.09 ± 0.03, 0.6 ± 0.4, 0.09 ± 0.03, 0.11 ± 0.04, 0.09 ± 0.03, and 0.08 ± 0.0 ng/mL, respectively. And the mean residence time were 0.9 ± 0.1, 1.8 ± 0.1, 4.3 ± 0.3, 5.7 ± 3.5, 4.4 ± 0.5, 4.7 ± 0.5, 4.3 ± 0.8, 3.0 ± 0.6 h, respectively. Compared with the normal control group, the PK behaviors of alkaloids, flavonoids, and iridoids in the inflammatory model exhibited a trend of continuous changes, including higher bioavailability, slower elimination, delays in reaching the *C*_max_ and longer substantivity [169]. In addition, there were a handful of PK investigations of couplet medicines from HLJDD. Pan et al. explored the differences in PK and antioxidant effect of RC–FG couplet medicine and HLJDD in MCAO rats, which have been scarcely reported [170]. In MCAO group, the *C*_max_ of RC–FG and HLJDD were 1.188 ± 0.162 mg/L and 1.44 ± 50.295 mg/L, respectively. The *T*_max_ of were 0.625 ± 0.137 h and 0.458 ± 0.188 h, and the mean residence time were 97.042 ± 34.642 h and 101.306 ± 81.211 h, respectively. The results illustrated that HLJDD, compared with RC–FG couplet medicine, had a better assimilation effect, higher peak concentration, shorter time to peak, slower elimination rate, and longer mean dwell time in the context of cerebral ischemia. In addition, the extremely low concentrations of gardenia acid and geniposide could not prevent the superoxide dismutase from returning to normal values. This phenomenon may be due to other ingredients such as flavonoids and alkaloids, which played a similar role as iridoids. It demonstrated that HLJDD exhibited the ability in treating cerebral ischemia through its three major constituents synergistically. In rat liver microsomes incubation system, total flavonoids and alkaloids extracts exibited strong inhibition on rat cytochrome P450 isoenzymes activities, while HLJDD aqueous extract and total iridoids extracts had moderate inhibition ability. Total flavonoids and alkaloids also exhibited significant inhibitory effect on P-glycoprotein activity as evidenced by the efflux of Rhodamine-123 with IC_50_ of 104.6 and 82.6 μg/mL. However, the HLJDD aqueous extract and total iridoids extracts showed weak and negligible inhibitory effect on P-glycoprotein activity, respectively [171]. For further studies of herb–herb interactions and human situation in vivo, PK studies involving human intestinal and liver microsome preparations should also be conducted.

Common analytical methods employed in PK studies usually need relatively large amounts of sample [172]. An indirect competitive enzyme-linked immunosorbent assay based on monoclonal antibodies against geniposide was developed and was successfully applied to study the PK of geniposide in HLJDD in mice [173]. Therefore, a technology with higher detection sensitivity would quite help in PK studies especially in small animals.

Compared with abundant data of pharmacology and chemical composition studies, PK studies cannot well support and interpret the pharmacological actions of HLJDD. On one hand, it is difficult to confirm the active components. Although some effective components such as berberine were known, their pharmacological effects cannot represent the whole TCM formula. Due to the limitations of analytical methods, on the other hand, simultaneous detection and analysis of all chemical components cannot be carried out. Therefore, it is necessary to combine with other methods. For example, according to the pharmacodynamic data, the main parameters are calculated to indicate changes in an active ingredient, a group of components, synergies between components, or interactions between metabolites.

## Conclusion

It is well known that TCM formula is a complex system and combinations can make the prescriptions more suitable for clinical application through herb–herb synergic interactions that improve pharmacological activities. Different from the methodology and philosophy of western medicine, TCM focuses on the overall functional state of the patients and the adjustment of their balance, which has aroused ever-increasing interest worldwide, especially for the treatment of complex diseases [174]. HLJDD, a classic TCM formula to clear “heat” and “toxins”, is an aqueous extract of four herbal materials, RC, RS, CP, and FG in a ratio of 3:2:2:3. Although the four herbs show unique activities with varying abilities respectively, synergistic functions are exhibited when combining them in an appropriate proportion.

In this view, we summarize the phytochemical, pharmacological and PK investigations of HLJDD. The potential bioactive constituents of this formula can be classified as alkaloids, flavonoids, and iridoids. Among them, berberine, baicalin, and geniposide are the representative ingredients. Containing numerous compounds, HLJDD exhibits pharmacological activities in various aspects, including anti-tumer, hepatoprotection, anti-inflammatory, anti-allergy, lipid-modulating, CNS diseases, anti-bacterial, and gut microbiota-modulating. The main differences between the PK profiles of primary ingredients in HLJDD and pure compounds are reflected in some important PK parameters. HLJDD tends to present higher *C*_max_, shorter *T*_max_ and better pharmacological effects than that in single drug or couplet medicine. These results demonstrate that the co-occurring components in HLJDD might interact with each other.

To further shed light on compositive principle and action characteristics of HLJDD, several obstacles that represent the common problems of TCM need to be conquered. Firstly, accurately annotating and understanding the classical literatures of the utilization of Chinese medicine formula combined with totally randomized blank controlled double-blind clinical trials would help to confirm the therapeutic effects and reveal the adverse reactions. Secondly, personalized medicine is the specific signature of TCM, according to which one formula might be adopted to treat different diseases with the similar syndromes. One kind of disease, however, might be treated with different formula due to variations in syndromes. Then, with the modern biological technologies and pharmacological approaches, the investigations on clinical syndromes and the following development of pre-clinical research system in cell or animal with consistent pathological features or biomarkers are expected to interpret of rationality and rule of compatibility of monarchs, ministers, assistants and ambassadors in the prescription of Chinese medicine, to reveal the concepts of TCM theories such as heat-clearing and detoxifying. Moreover, unlike Western medicine, therapeutic system of TCM is established directly on the clinical practices. But the complex constitutes of herbals make it hard to note the exact activating components and the interfered node of pathophysiological process. Abundant ingredients of herbs commonly bear the burden of therapeutic efficacy through activating or inhibiting different targets. High-throughput screening on the targets associated with the representative signaling pathway and further pharmacological assay on the synergistic action of those chemicals are required to explore interaction network between the multiple components and the multiple targets. Novel form of TCM formula appearing as several chemical preparations is believed to substitute the primary formula, which could be endowed with typical chemical, pharmacological and pharmacokinetic features. Furthermore, based on these digital database resources, the interactions between the chemicals and targets and relationship between the targets need to be analyzed via the system pharmacology, which favors the prediction of the potential activate components and the underlying targets or signaling pathways of TCM formulas. The following work performed to validate these literature mining results includes transcriptomics, proteomics, metabolomics, and rigorous biochemics and pharmacologics. Finally, the exploration is always on for TCM formulas, which promote the determination of pivotal components and uncover the interesting pathological mechanisms in the context of positive clinical therapeutic effects.

The studies of TCM formula are based on thousands of years of clinical medication experience, which provides a guarantee for the direction of basic research. The basic researches can simplify the formula and enhance the targeting and specificity in the treatment of certain diseases. On the other hand, combined with the PK analysis, basic researches can explore meaningful monomer compounds. In addition, on the basis of pharmacological effect evaluation and molecular mechanism analysis, basic researches can develop new therapeutic compounds when combining with chemical synthesis technology. Finally, it is also worth noting that many disease markers, discovered because of their exact clinical value, could play a role in TCM formula where the active ingredients are not well defined and the treatment mechanisms are not clear. Therefore, the development of new compounds targeting these markers will provide effective research ideas and reliability assurance for the development of new drugs.

## Data Availability

Not applicable.

## Abbreviations

HLJDD: Huang-Lian Jie-Du decoction
TCM: traditional Chinese medicine
RC: *Rhizoma Coptidis*
RS: *Radix Scutellariae*
CP: *Cortex Phellodendri*
FG: *Fructus Gradeniae*
T2DM: type 2 diabetes mellitus
AD: Alzheimer’s disease
PK: pharmacokinetics
eEF2K: eukaryotic elongation factor-2 kinase
AMPK: AMP-activated protein kinase
mTOR: mammalian target of rapamycin
NF-κb: nuclear factor-kappa B
NO: nitric oxide
LO: lipoxygenases
LPS: lipopolysaccharide
IL: interleukin
TNF-α: tumor necrosis factor-alpha
IκB-α: inhibitor-kappa B-alpha
MAPK: mitogen-activated protein kinase
Th: T-helper
GLP-1: glucagon-like peptide 1
CNS: central nervous system
MCAO: middle cerebral artery occlusion
Akt: protein kinase B
Ang: angiopoietin
*C. albicans*: *Candida albicans*
MIC: minimum inhibitory concentrations
NA: neuraminidase
